# Evaluation of color changes during stability studies using spectrophotometric chromaticity measurements versus visual examination

**DOI:** 10.1038/s41598-022-13025-3

**Published:** 2022-05-27

**Authors:** Lara-Malenka Sakiroff, Philip Chennell, Mouloud Yessaad, Bruno Pereira, Yassine Bouattour, Valérie Sautou

**Affiliations:** 1 CHU Clermont-Ferrand, Pôle Pharmacie, F-63000 Clermont-Ferrand, France; 2 Université Clermont Auvergne, CHU Clermont Ferrand, Clermont Auvergne INP, CNRS, ICCF, F-63000 Clermont-Ferrand, France; 3 CHU Clermont-Ferrand, Unité de Biostatistiques, DRCI, F-63000 Clermont-Ferrand, France

**Keywords:** Analytical chemistry, Drug development

## Abstract

Stability studies are essential to be able to assign an expiration date to medications. Color variation is one of the organoleptic characteristics of actives substances or medications which can indicate the presence of contaminations, impurities or degradations products. However there is no data available comparing the often used visual examination with spectrophotometric measurements during stability studies. The aim of this study was therefore to evaluate precisely how different the two methods are, by comparing the change of color of two drug formulations chosen as models, assessed by visual examination versus a spectrophotometric colorimetric analysis. Paracetamol and parenteral nutrition solutions were stored in stress conditions for up to 46 days, and were subjected to a visual examination using color reference solutions and to lightness and chromaticity measurement to determine their specific color by UV–Vis spectrophotometry. The color of paracetamol solutions changed faster when exposed to stress condition (light), as did the PNS when exposed to heat. In both cases, color variations were detected earlier and more precisely by UV–Vis spectrophotometry than by visual examination. Color measurement using an UV–Vis spectrophotometry should advantageously replace visual examination when assessing colors changes during drug stability studies.

## Introduction

Stability studies are essential to be able to assign an expiration date to medications, including compounded preparations, thus participating in guaranteeing their quality, security and efficacy. In order to be performed adequately, global guidelines, like those issued by the International Council for Harmonization of Technical Requirements for Pharmaceuticals for Human Use (ICH), were developed: of these, 6 guidelines (Q1A to Q1F) are devoted to stability studies^1^.

Among characteristics that must be evaluated during stability studies, color variation is one of the organoleptic characteristics of actives substances or medications which can indicate a presence of contaminations, impurities or degradations of products^2–6^. Stark et al*.* showed the existence of a good correlation between the apparition of a color and the chemical instability of the active substance of white solid dosage forms of captopril, flucloxacillin, cefoxitin and theophylline^7^. Likewise, Fairbrother et al*.* highlighted that a color change observed during nystatin accelerated stability studies was related to chemical decomposition and loss of antifungal activity^8^. Color is therefore an important parameter that must be controlled in order to help validate the quality, security and efficacy of medications and any variation must be checked for significance. Originally, the European Pharmacopeia described only a visual examination protocol for comparing tested solutions to color reference solutions (Monography 2.2.2: the degree of coloration of solutions^9^). However, this method is observer dependent and necessitates a well-trained operator, able to distinguish precisely the different coloration, as well as adequate lighting and examination conditions. Yet even when those prerequisites are met, visual examination remains a subjective control which gives variable results^10^. To alleviate this problem, ICH guideline QA6 recommends that a quantitative approach (for example using UV–Vis spectrometer) be used to precisely quantify color change during storage^11^. Furthermore, the US Pharmacopeia describes a method utilizing a UV-spectrometer to measure lightness and chromaticity to determine a specific color^12^, and this approach has also been integrated into the European Pharmacopeia in its latest edition^9^.

Despite these recommendations, most published stability studies (even those performed recently) fail to adequately integrate this notion, and still rely on visual examination to track potential color change. This could be possibly linked to incomplete knowledge about the limits of visual examination when compared to an analytical quantification. For example, the authors of these studies perhaps do not apprehend to what extent visual evaluation is imprecise, as there is no data available comparing exactly how different these examinations are.

The aim of this study was therefore to evaluate precisely how different the two methods are, by comparing the change of color of two drug formulations chosen as models (known to change color under stress conditions), assessed by visual examination *versus* a spectrophotometric colorimetric analysis.

## Materials and methods

### Materials

The following products were used during the study:

- Paracetamol 10 mg/ml B. Braun® ampoules (PA) were purchased from B. Braun (batch 19441417 and 20041405, expiring 09/2021 and 12/2021, B Braun, Melsungen, HE, Deutschland).
- The parenteral nutrition solutions (PNS) used in the study were produced in-house at the initiation of the study (composition described in Table 1) and conditioned in 125 mL simple layered Ethylene–vinyl acetate (EVA) bags for parenteral nutrition (ref E1301OLPF, batch I057CA, expiring 03/2024, ExactaMix®, Baxter, Zurich, Switzerland).
- Ready to use color reference solutions set of colors B (batch #BCCB4890 exp.: 04/2023) and set of colors Y (batch #BCCB2116 expiring 02/2023) conforming to monography 2.2.2 of the European Pharmacopoeia were purchased from Sigma-Aldrich (St Louis, MO, USA).
- Sterile deionized water and 0.9% sodium chloride was purchased from Fresenius Pharmaceutical Inc. (Sèvres, France).

**Table 1:** Composition of standard parenteral nutrition solution.

Components				Concentrations
Denomination	Batch number	Expiration date	Provider	
Glucose 50%	19I19E 19108B	09/2020	Macopharma	157.68 g/L
Vaminolact®	16NM6129	11/2021	Fresenius Pharmaceutical Inc. (Sèvres, France)	151.72 mmol/L
Sodium chloride 7.5%	19F027	03/2021	Assistance Publique-Hôpitaux de Paris (AP-HP, Paris, France)	10.04 mmol/L
Potassium chloride 7.46%	18F050	06/2021	AP-HP	22.02 mmol/L
Calcium gluconate 10%	4,402,557	12/2022	Aguettant (Lyon, France)	8.14 mmol/L
Magnesium sulfate 10%	19F032	03/2021	AP-HP	2.02 mmol/L
Phocytan® 0.33 mmol/mL	GOO52	01/2022	Aguettant	10 mmol/L
Water for injection	19I06C and 19B06B	09/2021 and 02/2021	Macopharma	Q.s*

*Q.s quantity sufficient.

### Study design

Paracetamol ampoules (PA) were subjected to either one of the following conditions whilst stored in a validated climate chamber (Binder GmbH, Tuttlingen, Germany) at 25 °C ± 1 °C, 60% ± 1% residual humidity:

- Exposure to UVA (0.60 ± 0.10 W/m^2^) and white radiations (average intensity of 6600 ± 200 lx).
- No exposure to light radiations (storage in the dark), protected in an opaque box.

PNS bags were stored in either one of the following conditions (both in opaque box to prevent any exposition to light):

- a validated climate chamber (Binder GmbH, Tuttlingen, Germany) at 25 ± 1 °C, 60 ± 1% residual humidity.
- a refrigerator at 5 ± 3 °C (Liebherr, Bulle, Switzerland) with daily temperature control.

At the initiation of the study and after 7, 14, 21, 28, 39 and 46 days of storage, 3 units of each type (PA and PNS) and for each condition were retrieved from storage and subjected to the following analyses:

- Visual examination.
- Chromaticity and lightness measurements.
- Specifically for the PNS: sterility test.

### Analyses

#### Visual examination

**Figure 1:**
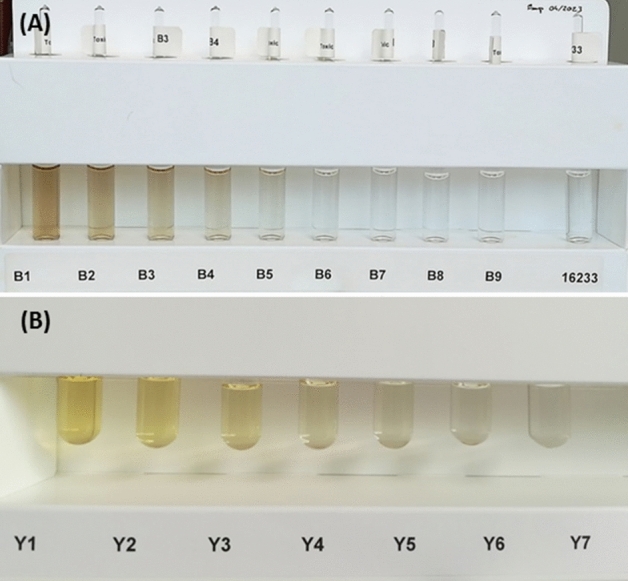
European Pharmacopeia color reference range solutions used for visual examination: (**A**) set of color B (brown) and (**B**) set of color Y (yellow).

Each sample was emptied into glass tubes and a visual exam was carried out in a double-blind randomized study. In brief, the tubes were blinded, then subjected to a visual examination performed by 3 trained technicians (none of whom had any known color vision deficiency, the test used by the Occupational Medical Service being Ishihara's Test for Color Deficiency), under illumination of a LED lamp diffusing white light (color rendering index of 85, 4000 K), and a white paper support to maximize color contrast. Room ambiance light was not controlled. The technicians were asked to classify the color level of the test solutions according to the reference samples containing 10 solutions ranging from B1 (brownest solution) to B9 (clearer solution) for the PA study or according to 7 solutions ranging from Y1 (yellower solution) to Y7 (clearer solution) for the PNS study (see Fig. 1). The classification was performed using the following protocol: the sample was compared to the least colored reference sample (B9 or Y7), and if the sample was equally or less colored than the reference sample this point it was considered to be of that value. If the sample was considered more colored than the reference point then it was compared to the following darker reference point (B8 or Y7) and the previous methodology was applied, and then so on. In order to mimic real-life visual examination during hospital stability-testing, all the samples could be handled at will by the technicians, and examined from any distance they found suitable. The technicians were given all the time needed to repeat their examination of the same 3 samples (triplicate analysis (3 units) for each time point per technician). The results given correspond to their final decision (average of the three evaluations for the three technicians).

The technicians had given their informed consent to conduct the visual examinations of the color of the contents of the glass tubes, and the study was checked for accordance and applicability with French law (2016–1537) concerning studies involving human subjects. The local research ethics committee (Committee for the protection of persons, *CPP Sud-Est VI of Clermont-Ferrand*), was also consulted and the study was approved under reference *2021/CE 42*.

#### Chromaticity and lightness measurements

**Figure 2:**
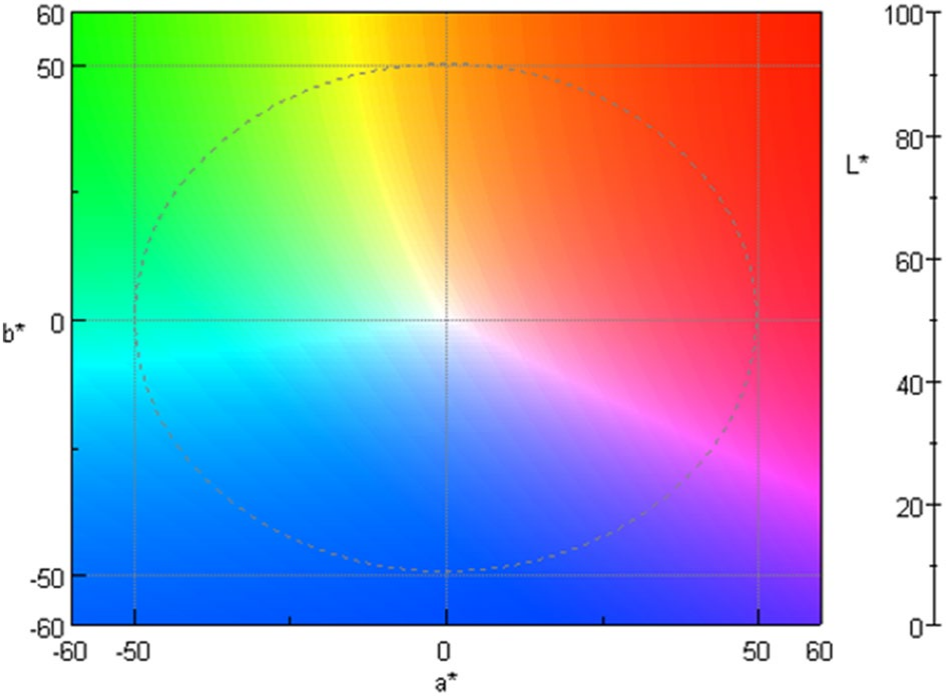
Representation of the CIE L*a*b* dimensional color space, where a* and b* represent the chromaticity and L* the lightness (White, L* = 100; Black, L* = 0).

Before analyzing the samples, a blank sample was realized with reference deionized water to obtain 100% of transmittance. Samples were transferred into the quartz cell previously cleaned twice with purified water and twice with the sample, then subjected to the color analysis. In this study, the CIE L*a*b* color space (L*, a*, b*) were used to represent the color changes^13, 14^. A representation of this color system is presented Fig. 2. These color parameters were measured using a Jasco V-670 spectrometer (Jasco Corporation, Lisses, France) with a quartz measuring cell. Transmittance spectra were obtained by using the following parameters: color system used was L*a*b* with 2 degrees of standard observers, a light source D65 (wave length between 780 and 380 nm, with the changeover wavelength between the deuterium and halogen lamps set to 340 nm), data pitch 5 nm, color matching JIS Z8701-1999, scan speed 1000 nm/min, UV/visible bandwidth 2 nm. Color analysis of spectra were performed using the “Color diagnosis” software, version 2.2.0.1 (JASCO Corporation).

The calculated color difference (∆E) was calculated using the following equations for the spectrophotometric analysis^15^:

1 $$\Delta {\text{a}}^{*} = {\text{ a}}^{*} - {\text{a}}_{0}^{*}$$

2 $$\Delta {\text{b}}^{*} = {\text{ b}}^{*} - {\text{b}}_{0}^{*}$$

3 $$\Delta {\text{L}}^{*} = {\text{ L}}^{*} - {\text{L}}_{0}^{*}$$

4 $$\Delta {\text{E }} = \, \left( {\left( {\Delta {\text{L}}^{*} } \right)^{2} + \left( {\Delta {\text{a}}^{*} } \right)^{2} + \left( {\Delta {\text{b}}^{*} } \right)^{2} } \right)/0.{5}$$

a_0_*, b_0_* and L_0_* were the initial values at day 0 (immediately after sample preparation) before exposition to stress condition (light exposition or ambient temperature), and ∆a*, ∆b* and ∆L* were the difference in chromatic coordinates and lightness.

The values of each color parameter are expressed as the mean of three measurements on three different samples. The values used for the “visual examination” analysis are the corresponding value of chromaticity and lightness for the selected colors reference solutions, which had been previously measured by the UV–Vis spectrometer (see Supplementary Materials File S1).

#### PNS: sterility test

The sterility test was carried out on parenteral nutrition bags by membrane filtration technique as recommended by monography 2.6.1 “Sterility” of European Pharmacopeia. This test was carried out under class II microbiological safety cabinet Faster BH-2004 (Faster Srl., Ferrara, FE, Italy) equipped with vertical laminar flow. One bag was passed by 0.45 μm pore Filter Funnel (ThermoFischer, Waltham, MA, USA) and rinsed with 250 mL of NaCl 0.9%. The sterility test required two liquid culture media: tryptone soya broth USP (batch: 2972097 and exp: 04/2021 ThermoFischer, Waltham, MA, USA) and thioglycollate medium USP (batch 2970622 and exp.: 07/2020 ThermoFischer, Waltham, MA, USA). The culture media were observed after 14 days of incubation.

#### Statistical analysis

Statistical analyses were performed using Stata software (Version 15, StataCorp, College Station, US). Continuous data were expressed as mean and standard-deviation. The assumption of normality was assessed using the Shapiro–Wilk test. To compare the change of color of two drug formulations chosen as models (paracetamol solutions and PNS) during stress conditions to induce instabilities, random-effects models for repeated data were performed. The “observer” was considered as random-effect in order to measure between and within observer variability (several measures for each observer) whereas groups (visual examination vs. spectrophotometer and with or without stress conditions), time and groups x time interaction were fixed effects. The normality of residuals from these models was studied as aforementioned. When appropriate, a logarithmic transformation was applied to access the normality of dependent variables. The tests were two-sided, with a type I error set at 5%. A Sidak’s type I error was applied to take into account multiple comparisons.

## Results

### Paracetamol ampoules

**Figure 3:**
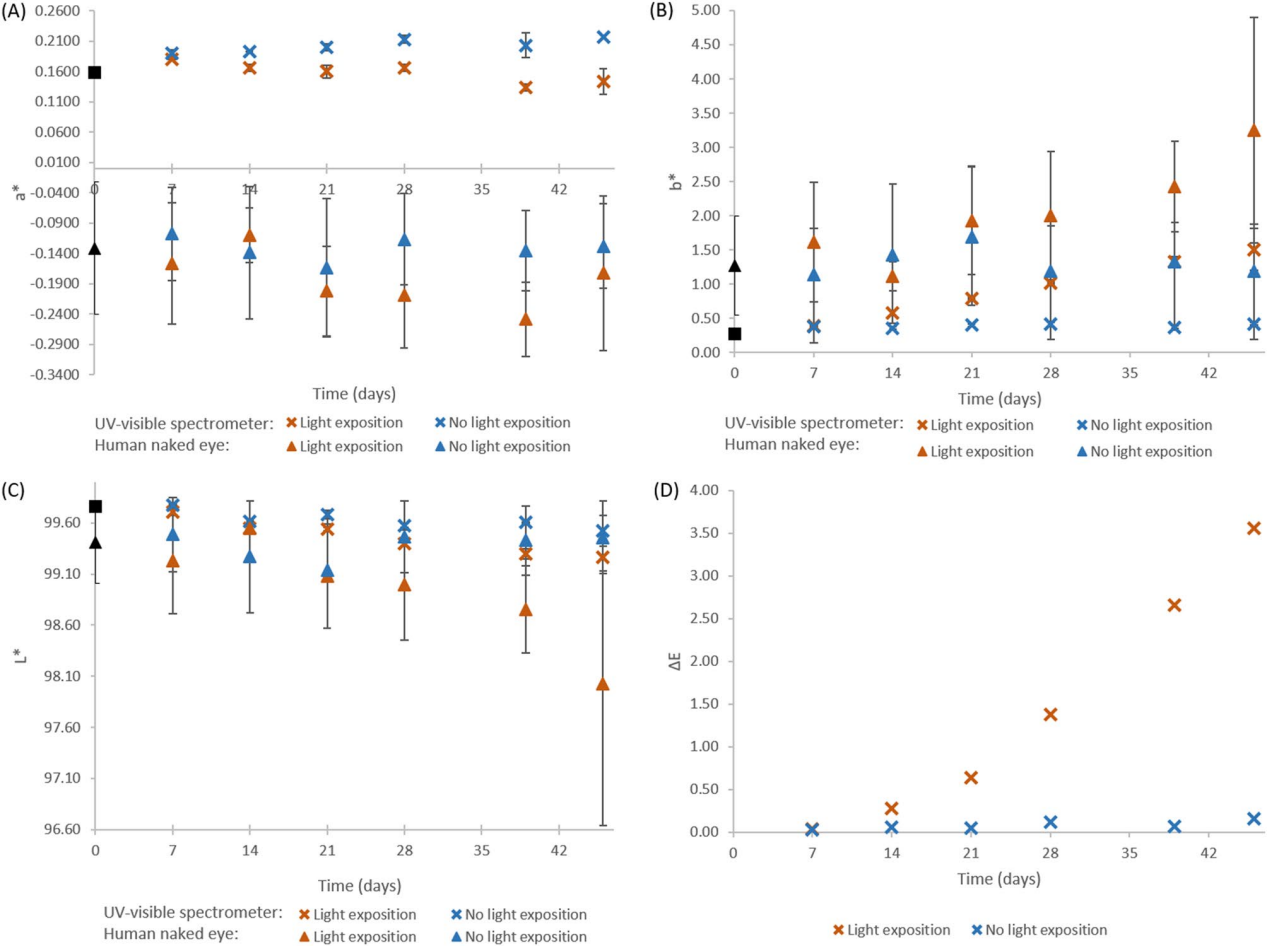
Colors parameters of paracetamol solution observed by UV–Vis spectrometer or visual examination according to time and light exposition conditions: (**A**) a*, (**B**) b*, (**C**) L* and (**D**) ∆E (only for the spectrophotometric analysis). Results expressed as mean ± standard deviation, n = 3, except for (**D**) (difference of means, n = 1). Crosses: spectrophotometric data; triangles: human naked eye (visual examination) data.

Figure 3 presents the evolution of each chromaticity parameters (a*, b* and L*) and difference of perception (ΔE) calculated using Eq. (4). Complete data is presented in Supplementary Data File S1. Chromaticity measurements at day 0 were of a* and b* of 0.172 ± 0.027 and 0.278 ± 0.026, respectively (mean ± standard deviation), thus confirming the slight very slight pinkish color detected by the visual examination. Overall, throughout the study and for both storage conditions, a* remained globally stable, b* values increased and L* values decreased, indicating to a shift toward redder and darker solutions. For parameter b*, even if the spectrophotometer detected a change from day 14 onwards (p < 0.001), the values increase faster when the samples were exposed to light compared to when kept in the dark (1.507 *versus* 0.420 after 46 days). The change was also significant after 14 days for parameter L*, for both exposition conditions (p < 0.001 and 0.004 for respectively with and without light exposition). Comparatively, visual examination did not detect any significant change of chromaticity or lightness throughout the study when the sample were stored in the dark (p-values of 0.838, 0.598 and 0.578 for respectively a*, b* and L* after 46 days of storage). When the samples were exposed to light radiations, the difference still wasn’t significant after 28 days for a* (p = 0.054), but it became so after 39 days (p = 0.002). b* differences were however slightly significant for the visual examination from day 28 onward (p = 0.002), but a change in L* vales was only detected as significant after 39 days (p = 0.015). ΔE values varied from 0.027 to 0.163 for the samples stored in the dark (days 7 to 46) and from 0.035 to 3.558 (days 7 to 46) when exposed to light. In that condition, ΔE values were of 1.381 and 2.653 at days 28 and 39, respectively.

As for the study of the parenteral nutrition solutions, the visual examination results are more variable than the measurements made by the spectrophotometer.

### Parenteral nutrition solution

**Figure 4:**
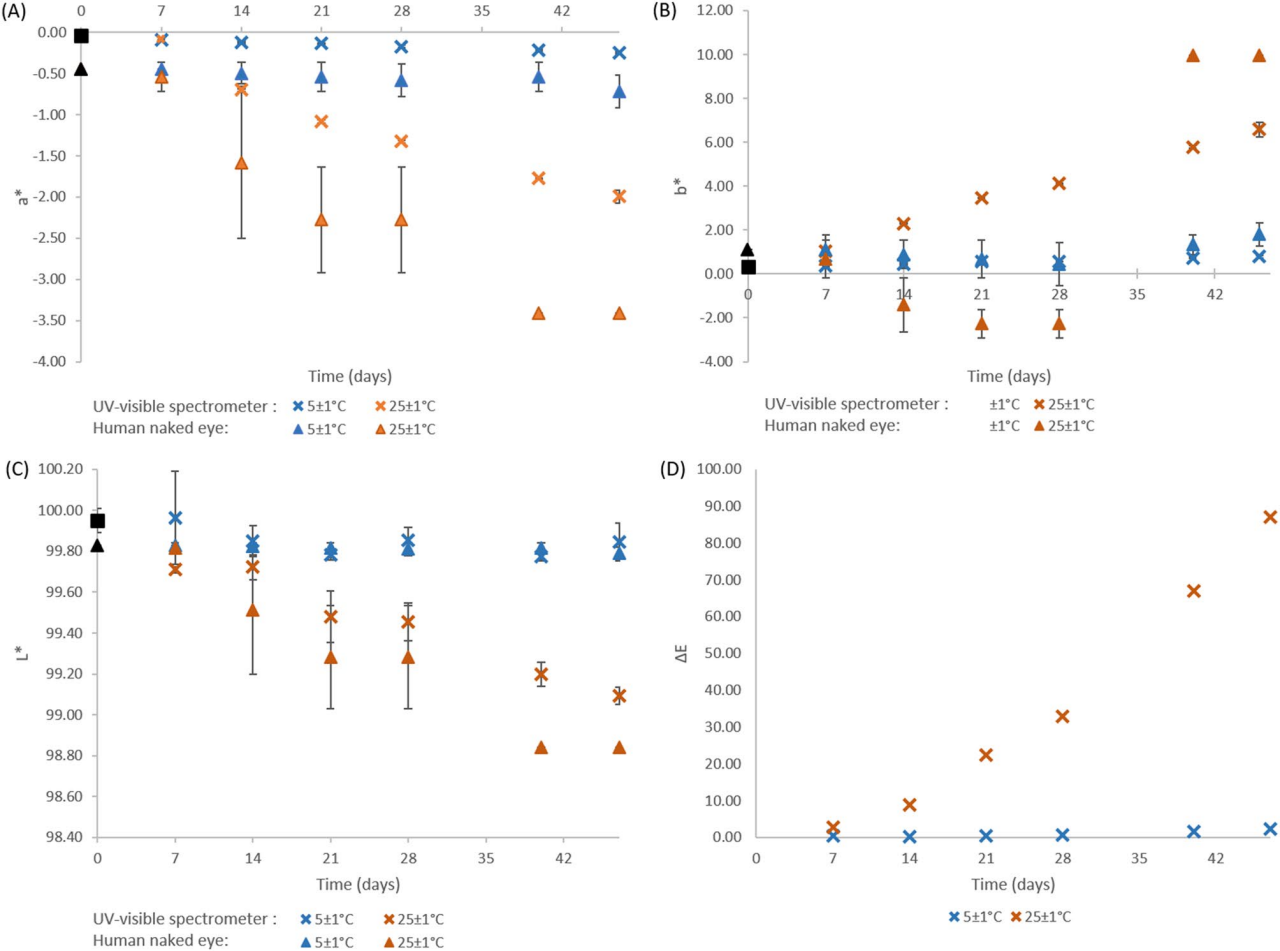
Colors parameters of parenteral nutrition solution observed by UV–Vis spectrometer or visual examination according to time and light exposition conditions: (**A**) a*, (**B**) b*, (**C**) L* and (**D**) ∆E (only for the spectrophotometric analysis). Results expressed as mean ± standard deviation, n = 3, except for (**D**) (difference of means, n = 1). Crosses: spectrophotometric data; triangles: human naked eye (visual examination) data.

Figure 4 represents the evolution of each chromaticity parameters (a*, b* and L*) and difference of perception (ΔE) calculated by Eq. (4). Complete data is presented in Supplementary data file S1. Chromaticity measurements at day 0 were of a* and b* of − 0.045 ± 0.008 and 0.310 ± 0.093, respectively (mean ± standard deviation), thus confirming the slight yellow color detected by the visual examination. Overall, throughout the study and for both storage conditions, a* and L* values decreased and b* values increased, corresponding to a shift toward browner and darker solutions. The spectrophotometer detected a change of chromaticity components a* and b* for both storage conditions (5 °C and 25 °C) after only 7 days of storage (p < 0.0015), but a change of lightness was only detected after 21 days when the PSN solutions were stored at 5 °C. Comparatively, the visual examination detected a change (chromaticity of lightness) after 14 days (25 °C storage condition, p < 0.001) or 46 days (5 °C storage condition, p < 0.018). ΔE values varied from 2.83 to 86.9 at 25 °C (days 7 to 46) and from 0.41 to 2.02 (days 7 to 46) when stored at 5 °C, with a ΔE value of 1.40 at day 39. Overall, it can also be noticed that the visual examination results are more variable than the measurements made by the spectrophotometer.

### Sterility test

Parenteral nutrition solution sterility test came back negatively after 14 days of incubation and allows us to exclude a microbial contamination on our batch and therefore any microbial cause to the change of color). The paracetamol solutions were already conditioned in sterile ampoules by the manufacturer, and were discarded after analysis.

## Discussion

In this study assessing the change of color of two different drug formulations in stress conditions, assessed by visual examination and spectrophotometric measurements, the results showed that the spectrophotometric analysis was systematically capable of detecting a change of color well before visual examination, especially when the changes were subtle (paracetamol solutions).

For the study, the two drug formulations that were chosen (paracetamol solutions and parenteral nutrition solutions) are well known for their propensity to change color when stressed. Paracetamol is a widely used drug but is susceptible to photodegradation. This phenomena seems to be caused by hydrolysis and oxidation accelerated by radiation exposition. Particularly, the breakdown of paracetamol to quinonimine and related compound leads to a gradual change of color through pink to brown by oxidative degradation in solid paracetamol, while its degradation when solubilized seems to be caused by acid and base-catalytic hydrolysis reaction and leads to what has been described as initially a pink color that darkens to brown over time^16–18^.

PNS containing amino-acids are also known to be prone to change of color, and become browner with time, especially at ambient temperature. This phenomenon is generally attributed to Maillard’s reaction and the degradation of the amino acids they contain, especially lysine, glycine and methionine^19^. Nonetheless, Yailian et al. showed that PNS became first more yellow than brown, and linked it to the increased presence of cysteine, which is the oxidized dimer form l-cysteine^20^. This information validates the use of the European Pharmacopoeia set of colors B (brown) and Y (yellow) as references for the visual examination for respectively the paracetamol and PNS formulations.

As expected, both drug formulations changed color during the study when exposed to their stress condition (light for the paracetamol solutions and ambient temperature for the PNS, which remained sterile for storage conditions). In both cases (stressed drug formulations), the spectrophotometric analysis objectivized a change of color and lightness before it was detected by visual observation, with a difference of 2 weeks for the paracetamol solutions (change of color detected at day 14 versus day 28 at the best) and 1 week for the PNS (change of color detected at day 7 versus day 14), for respectively the spectrophotometer analysis and the visual examination. Interestingly, when the solutions were stored if optimum conservation conditions (in the dark for the paracetamol solutions and at 5 °C for the PNS), the difference in detection capacity was even more flagrant. When the spectrophotometric analysis detected a change after 14 and 7 days (for respectively the paracetamol solutions and PNS), the visual examination either didn’t notice any change (paracetamol solutions), or only noticed the change after 46 days of storage. These findings are consistent with the computed ∆E value, which expresses the difference of color perception. It has been proposed that when ∆E < 1, observers do not notice the difference, and for 1 < ∆E < 2 only experienced observers can notice the difference^21^, even if this general simplification might not be accurate for all colors, as variations have been noted depending on the original chromaticity during forced degradation studies^22^. Also, other authors have proposed other limits: Faghihi et al.^23^ indicates that ΔE < 1: undetectable with the human eye, 1.0 < ΔE < 3.3: distinguishable by a skilled individual, and ΔE > 3.3: easily observable with naked eyes, whilst Gupta et al.^24^ uses even wider acceptability thresholds (ΔE > 3.7-easily visible difference, ΔE between 3.7 and 1 acceptable difference, ΔE < 1-difference clinically not visible). In this work, for the paracetamol solutions, ∆E values stayed well beneath 1 (maximum value of 0.163) when the solutions were stored in the dark, indicating that the observers shouldn’t be able to notice a difference, which was confirmed by the results (no difference, p > 0.5 for a* and b*). When exposed to the light, ΔE values were of 1.381 and 2.653 at days 28 and 39, respectively, and it was at those days that the visual examination did just begin to notice a difference. This finding was also valid for the PNS: ΔE values were of 1.39 at 39 days and 2.02 after 46 days at 5 °C, and the change of color was detected by the observers at day 46 (but not at day 39). However, for the 25 °C storage condition, ΔE was of 2.8 after 7 days, thus suggesting an easily detectable color change, yet the change was not detected at day 7 (p = 0.68 and 0.14 for a* and b*, respectively) but only at day 14 (p < 0.001 for a* and b*).

The increased variability that was noticed for the visual examination results could be explained by several factors, one of them possibly being differences in perception. This variability is in inherent to any visual examination. The use of a randomized double-blind system meant that the observers were not capable of identifying any of the tubes, thus guarantying that they were not influenced in any way. However, the correspondence of the visual observation with comparable values of a*, b* and L* was limited by the reference range solutions B and Y of the European Pharmacopeia, as there were only 9 and 7 points respectively in the colors sets. Observers which saw some intermediate colors between two color levels (for example between Y6 and Y5) had to choose a level of the solution range. This method of evaluation could have limited the personal perception of each observer, and is one of the study’s limitations. Also, despite the visual examination having been standardized (visual examinations performed under the illumination of a LED lamp diffusing white light of 4000 K), the room ambiance light in which the study was performed was not standardized. Differences of the ambient light (linked for example to different weather conditions) could also impact color perception of the technicians and reduce their capacity to precisely attribute the color of the solutions. Unless this parameter is carefully controlled, it is another limitation of any visual examination performed for the color assessment during stability studies. A sample size of three (like what was used in this work) is of common use in several scientific areas, including practical stability studies (see Bardin et al.^25^ and the recommendations issued jointly the French Society of Clinical Pharmacy (SFPC) and European Society of Hospital Pharmaceutical Technologies (GERPAC)^26^). However, like all sampling it can incur bias. It is possible that the number of samples was insufficient to allow ideal color evaluation by the examinators (also possibly explaining the variability that was observed for the visual examination results), however as this is representative of real-life situations it only serves to reinforce the case for the use of a spectrophotometric color analysis during practical stability studies. In this study, the CIELab formulas were used, as opposed to those of the CIEDE2000 color difference formulas. Using the latter could possibly have resulted in a better fit than with the CIELAB formulas, however it seems unlikely that the differences would impact the interpretation of our results. Indeed, when comparing the CIELab and CIEDE2000 color difference formulas, the work published by Gomez-Polo et al.^27^ indicates that although the coefficients of linear correlation estimating the color differences with the CIELAB and CIEDE2000 formulas comparatively with perceived colors of the participants were higher with CIEDE2000 than with CIELAB, they remained similarly low (0.289 and 0.176, respectively). Also, the CIEDE2000 is considerably more complex to use^28^, and could result in erroneous interpretations if used during practical drug stability tests.

Drug stability studies generally include a visual examination criteria, during which the observer must note any change of aspect, and research potential haziness, presence of visible particles and color change^11, 25, 26^, but do not include any definition of a maximum allowed color change, possibility because of the limits of usual visual examination. However, few authors include a spectrophotometric analysis of the potential color change, and rely on visual subjective evaluations, therefore potentially missing early signs of instabilities. Indeed, to the best of our knowledge, it seems that only three recent works investigating the stability of drugs included this analysis in their study. Won et al. proved that pemetrexed degradation was linked to a color change to yellowish solutions, confirmed by increasing values of b*, and that adding different antioxidants to the formulation not only decreased various breakdown products but also limited the variation in b* values^29^. Chennell et al. also showed that the evolution of color towards the red measured by colorimetry of amphotericin B solutions happened in parallel to a decrease in amphotericin B concentrations. However, the appearance of a breakdown product (assessed by a stability indicating method) did not induce any color change for atropine solutions stored at 25 °C^30^. Colorimetric analysis could also indicate the presence of other compounds, for example like toxins^31^. Overall, spectrophotometric colorimetric assessment of color change is another tool, simple to implement, which could help evaluate the stability of drugs during stability testing.

## Conclusions

This work has shown that a colorimetric analysis should replace visual examination for color determination and research of color change during stability studies, as this analysis can detect changes more precisely and earlier than by visual examination, in both chromaticity and lightness.

## Supplementary Information

Supplementary Information.

## Data Availability

All the raw data pertaining to this work is available in the Supplementary Information file.
