# Supplementary material for: Evaluation of color changes during stability studies using spectrophotometric chromaticity measurements versus visual examination
Source: Sci Rep. 2022 May 27;12:8959. doi: 10.1038/s41598-022-13025-3 (PMC9142492; doi:10.1038/s41598-022-13025-3)
Supplement: Supplementary file 1 — Supplementary Information. [file 41598_2022_13025_MOESM1_ESM.zip › Supplementary materialsR1b/File S1.docx]

**Evaluation of color changes during stability studies using spectrophotometric chromaticity measurements versus visual examination**

Lara-Malenka Sakiroff^1^, Philip Chennell^2^*, Mouloud Yessaad^1^, Bruno Perreira^3^, Yassine Bouattour^2^ and Valérie Sautou^2^

^1^ CHU Clermont-Ferrand, Pôle Pharmacie, Clermont-Ferrand, France

^2^ Université Clermont Auvergne, CHU Clermont-Ferrand, CNRS, SIGMA Clermont-Ferrand, ICCF, Clermont-Ferrand, France

^3^ Unité de biostatistiques (délégation à la recherche clinique et à l’innovation), CHU Clermont-Ferrand, 63000 Clermont-Ferrand, France

* Correspondence: [pchennell@chu-clermontferrand.fr](mailto:pchennell@chu-clermontferrand.fr)

The representation of chromaticity (a* and b*) and lightness (L*) for the colors reference solutions B (B1 to B9) and Y (Y1 to Y7) measured by the UV-visible spectrometer are presented Figure 1.


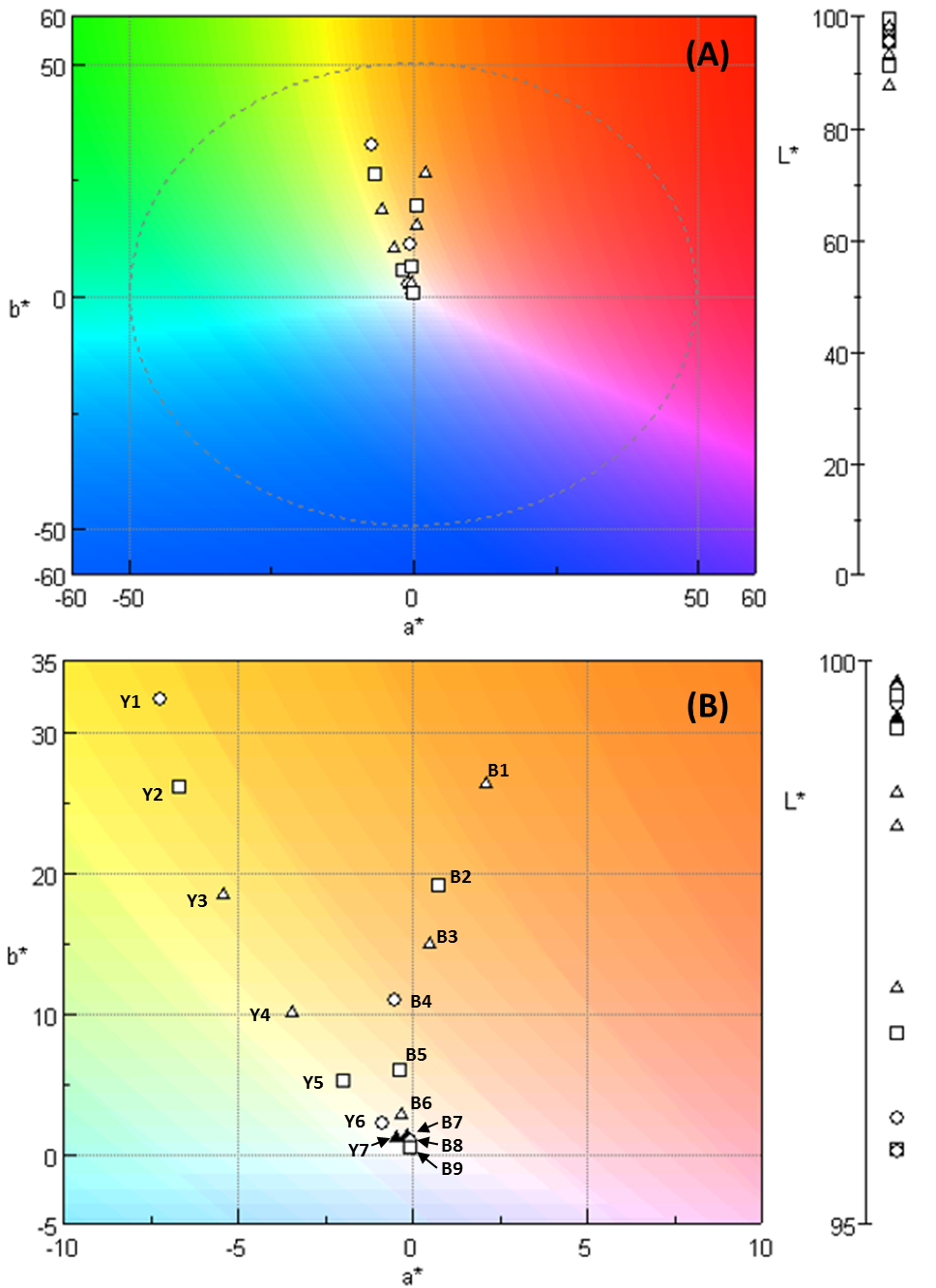


**Figure 1:** Chromaticity and lightness values for the colors reference solutions Y (Y1 to Y7) and B (B1 to B9). (B) is a close-up view of (A).
